# Channel State Information from Pure Communication to Sense and Track Human Motion: A Survey

**DOI:** 10.3390/s19153329

**Published:** 2019-07-29

**Authors:** Mohammed A. A. Al-qaness, Mohamed Abd Elaziz, Sunghwan Kim, Ahmed A. Ewees, Aaqif Afzaal Abbasi, Yousif A. Alhaj, Ammar Hawbani

**Affiliations:** 1 School of Computer Science, Wuhan University, Wuhan 430072, China; 2 Department of Mathematics, Faculty of Science, Zagazig University, Zagazig 44519, Egypt; 3 School of Electrical Engineering, University of Ulsan, Ulsan 44610, Korea; 4 Department of Computer, Damietta University, Damietta 34511, Egypt; 5 Department of Software Engineering, Foundation University, Islamabad 46000, Pakistan; 6 School of Computer Science and Technology, Wuhan University of Technology, Wuhan 430070, China; 7 School of Computer Science and Technology, University of Science and Technology of China, Hefei 230072, China

**Keywords:** RSSI, CSI, Wi-Fi, human activity recognition (HAR), device-free

## Abstract

Human motion detection and activity recognition are becoming vital for the applications in smart homes. Traditional Human Activity Recognition (HAR) mechanisms use special devices to track human motions, such as cameras (vision-based) and various types of sensors (sensor-based). These mechanisms are applied in different applications, such as home security, Human–Computer Interaction (HCI), gaming, and healthcare. However, traditional HAR methods require heavy installation, and can only work under strict conditions. Recently, wireless signals have been utilized to track human motion and HAR in indoor environments. The motion of an object in the test environment causes fluctuations and changes in the Wi-Fi signal reflections at the receiver, which result in variations in received signals. These fluctuations can be used to track object (i.e., a human) motion in indoor environments. This phenomenon can be improved and leveraged in the future to improve the internet of things (IoT) and smart home devices. The main Wi-Fi sensing methods can be broadly categorized as Received Signal Strength Indicator (RSSI), Wi-Fi radar (by using Software Defined Radio (SDR)) and Channel State Information (CSI). CSI and RSSI can be considered as device-free mechanisms because they do not require cumbersome installation, whereas the Wi-Fi radar mechanism requires special devices (i.e., Universal Software Radio Peripheral (USRP)). Recent studies demonstrate that CSI outperforms RSSI in sensing accuracy due to its stability and rich information. This paper presents a comprehensive survey of recent advances in the CSI-based sensing mechanism and illustrates the drawbacks, discusses challenges, and presents some suggestions for the future of device-free sensing technology.

## 1. Introduction

In recent years, human localization, human motion detection, and Human Activity Recognition (HAR) have gained more attention due to rapid advancements in the fields of computing and sensing techniques that can be applied in different applications, such as Human–Computer Interaction (HCI), e-gaming, gesture recognition, and surveillance, etc. [1]. Human activity recognition (HAR) is a field of computing research associated with human motion and activities in a controlled environment. The motion can be analyzed through the data collected from a combination of reasoning and sensing techniques to provide personalized support in various applications [2]. The conventional HAR methods are used in different applications, such as eldercare, healthcare, entertainment, and security [3,4]. However, traditional approaches are often considered device-based techniques, as they use various sensing devices such as cameras (vision-based) [5,6], acoustic sensors [7,8], accelerometers [9,10], wearable sensors [11,12], environment installed sensors [13,14], and smartphones [15,16]. Table 1 shows some previous HAR studies that used different devices and software-based solutions to obtain human action recognition.

Vision-based methods often require installing monitoring cameras in the field of interest to capture human activities. Therefore, vision-based methods require good lighting conditions. The research in vision-based sensing is restricted because cameras cannot see through walls. Sensor-based methods can be classified into different categories as illustrated in Table 1. Each category has a specific drawback. Sensor-based techniques require burdensome installation in the perceived environments or on the target human bodies.

**Figure 1:**
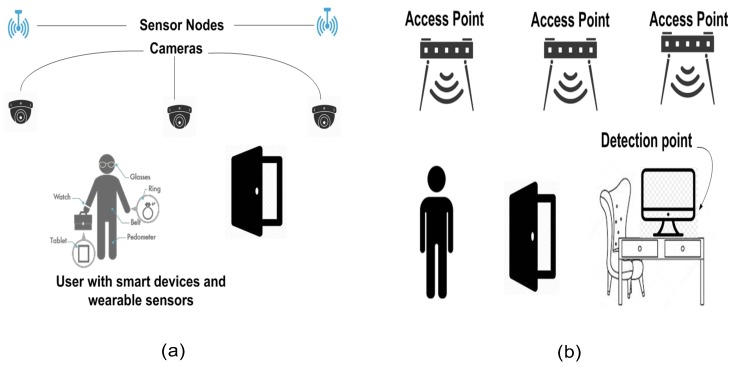
The differences between device-free and device-based sensing mechanisms; (**a**) device-based mechanisms; (**b**) device-free mechanisms.

Wireless sensing technology is a new sensing mechanism that does not require sensor devices to be installed or attached to the target object. It is also known as a device-free sensing technology. Figure 1 shows the main difference between device-based and device-free sensing mechanisms. As shown in Figure 1a, the device-based sensing mechanisms require burdensome installations either in the test environment or on the target object. In the device-free sensing mechanisms, the target object is free from sensing devices as the test area has no sensor devices (see Figure 1b).

The shadowing effects caused by human targets moving in the line-of-sight (LOS) i.e., the area between the transmitter and the receiver of an indoor environment can be exploited for tracking of human motion in closed environments [17,18]. The Received Signal Strength Indicator (RSSI) of wireless signals are adversely affected by object movement in indoor environments. Thereby, such variability can be leveraged to track human movement and activities in closed environments without installing additional equipment. Such observations had opened a new sensing mechanism that relies on ubiquitous wireless devices that are available everywhere (i.e., Wi-Fi devices).

In the last decade, some RSSI-based approaches had been presented for human localization [19,20,21,22,23,24,25,26,27] and human motion detection [28,29,30,31,32]. RSSI-based mechanisms, unlike Radio Tomographic Imaging (RTI), use several radio signal reflections to localize objects and track movement and activities [33,34,35]. However, RTI-based methods require installation of a large number of sensors for tracking purposes. RSSI-based technologies have been used to classify human actions, such as standing, lying, walking, sitting, and other body mechanics [36,37,38,39]. Furthermore, RSSI technology can also be extended to classify micro-activities, such as human hand gestures [40,41] and breath rate estimation [42,43].

RSSI-based mechanisms have certain limitations because of the instability of RSSI caused by the environment variations that lead to spurious detections. The new trend metric in device-free motion detection based on Channel State Information (CSI) has attracted more attention during recent years. The open source CSI-Tool is presented by Halperin et al. [44,45] which enabled CSI to be exported from commodity wireless Network Interface Controllers (NICs). The CSI Tool is built on the Intel Wi-Fi Wireless Link (IWL) 5300 NIC (Intel Corporation, Santa Clara, CA, USA). Previous studies show that CSI outperforms RSSI in indoor human localization and motion detection. CSI achieves a localization accuracy less than one meter (m), whereas RSSI achieves a localization accuracy more than one meter [46].

CSI can detect human motion based environment changes. It reflects the varying multipath reflection induced by the existence of moving objects due to its frequency diversity [47]. Compared to RSSI, CSI is fine-grained channel information, whereas RSSI is coarse-grained channel information. RSSI channels measuring criteria is based on packets, whereas the Orthogonal Frequency-Division Multiplexing (OFDM) measurement is based on packet basis. Hence, RSSI is the average value of the received signals, whereas CSI is measured per OFDM subcarrier. CSI has both amplitude and phase information. RSSI performance is often affected by multipath effects. By observing the variation in CSI caused by body movements, it has been shown that, in static environments, CSI delivers better results when a movement occurs [48]. Researchers proposed numerous CSI-based sensing approaches including localization [49,50,51,52,53,54,55,56,57,58,59,60], tracking human movements [61,62,63,64,65,66], and crowd counting [67]. CSI has been extended to sense human macro-activities, such as sitting, standing walking, and falling [68,69,70,71,72,73,74,75,76,77], and micro-activities such as gesture recognition [78,79,80,81], keystroke recognition [82], lip motion using special devices [83], and sleep and breath monitoring [84,85,86,87].

Nowadays, Wi-Fi-based human sensing has gained wider popularity and attention. Researchers have presented many studies in this field. This study presents a comprehensive survey on the state-of-the-art studies of the CSI-based mechanism, including human localization, motion detection, and activity recognition. Additionally, it describes the recent advances in RSSI and Software Defined Radio (SDR) mechanisms. Furthermore, we discuss the current challenges and address some suggestions to improve Wi-Fi-based sensing methods to be able to implement them widely in the future.

The main contributions of this study can be summarized as follows:

- We present a comprehensive survey on the new emerging sensing technology called a device-free CSI-based sensing mechanism.
- We address the current advances in device-free CSI-based sensing techniques, summarize previous studies, highlight possible applications, and show achieved results.
- We highlight current limitations and challenges that still need further investigation to enhance device-free CSI-based sensing mechanism.

The remainder of this study is organized as follows. Section 2 describes previous Wi-Fi-based sensing mechanisms and reviews the existing approaches, including RSSI-based mechanisms and Wi-Fi radar or so-called Software Defined Radio (SDR) mechanisms. CSI-based mechanisms and proposed approaches are described in Section 3. Section 4 describes the CSI sensing methodology. Section 5 discusses the current challenges and future suggestions. Finally, Section 6 concludes this study.

## 2. Previous Wi-Fi-Based Sensing Mechanisms

### 2.1. Received Signal Strength Indicator (RSSI)

In device-free sensing, the RSSI measurement takes signal power into a known channel fading model to estimate the transmitter–receiver distance. The signal power decreases as the propagation distance increases, which results in signal fading. Thus, the received signal power can be leveraged to estimate the transmitter–receiver distance. The RSSI can be estimated as the following expression:

(1) $$P\left(r\right)=P_{0}-10_{n}log_{10}r/r_{0},$$

where P(r) is the received signal power (dB) measured at a distance *r*; $P_{0}$ is the received signal power measured at a reference distance $r_{0}$.; and *n* denotes the path loss exponent.

During the last decade, RSSI has been leveraged in vast device-free approaches, since RSSI features are available in almost wireless devices. Hereby, in this section, we summarize the pioneering studies as follows.

#### 2.1.1. RSSI-Based Localization and Motion Detection

Recently, RSSI has been exploited in various device-free human localization systems such as [19,20,21,22,23,24,25,26,27], and improved for tracking human motion [28,29,30,31,32]. Furthermore, it has been extended to classify human macro-activities such as [36,37,38,39], and human micro activities such as [40,41,42,43]. RSSI captures the propagation attenuation of Wi-Fi signals to track human motion. However, RSSI faces performance degradation in complex indoor environments that may lead to false detections [88].

In the last decade, Wi-Fi RSSI was a hot topic for research, where many studies were conducted to study the tracking of human motion in indoor environments. RSSI has been utilized in human localization, human motion detection, and activity recognition.

In [28,29,30,31,32], a number of motion detection methods have been presented. In these studies, the authors used RSSI to capture the test area variation that becomes anomalous when human motion occurs in the test area.

In [28], a human motion detection method is presented by leveraging the variation of RSSI standard deviation between a stationary access point (a transmitter) and a detection point (a receiver) at pre-defined positions. In [29,31], RSSI is used to capture the environmental variations that are caused if a human enters into the test environment. In [30], the authors proposed an RSSI-based intrusion tracking method, which can recognize several human intrusion patterns. Huang et al. [89] presented a person detection system based on RSSI, namely *WiDet*. The key idea of *WiDet* is to track pedestrian speed changes since each speed has a unique impact on the RSSI. A Convolutional Neural Network (CNN) was applied to identify several users and achieved an average detection rate of 94.5%. Additionally, Jun et al. [90] applied a solution metric to improve RSSI indoor localization mechanism, namely AP-Sequence, which splits the test area into a number of regions, each one is identified by a unique AP-sequence. It handles RSSI temporal fluctuations and devices’ heterogeneity using the relative RSSI differences between different access points. In [91], an RSSI-based positioning method is presented by leveraging the average number of RSSI from selected Wi-Fi access points. In [92], a comparison between RSSI of Bluetooth low energy (BLE), Wi-Fi, long-range wide area network, and Zigbee have been implemented. The results showed that Wi-Fi outperforms other mechanisms in location accuracy which achieved a localization accuracy of 0.664 m.

#### 2.1.2. RSSI-Based Macro-Activity Recognition

The RSSI mechanism has been utilized to classify human macro-activities (i.e., standing, lying, walking, sitting, etc.) [36,37,38,39]. In [36], the authors presented a device-bound and device-free HAR scheme based on the variation of RSSI in the test area. The proposed scheme classifies four macro-activities namely, sitting, lying, standing, and walking. However, the proposed system uses 802.15.4 RSSI to track activities with two scenarios. The first scenario is called a device-bound scenario because the human user is asked to carry a wireless node. The second one is called device-free scenario where the human user is asked to implement activities in wireless sensor networks without carrying a node. It achieves an average accuracy of 89% for the second scenario, and 88% by using accelerometers.

In [37,38], the authors presented human macro-activity recognition methods by measuring changes of wireless signals induced by human motion in the test area. Several human activities have been classified (walking, standing, crawling and lying) and achieved an acceptable accuracy in different scenarios. However, the proposed method was implemented by using SDR with the Universal Software Radio Peripheral (USRP).

In [39], a Wi-Fi-assisted HAR method is presented. This method uses data mining techniques to abstract RSSI fingerprints of several human activities. The proposed scheme tests three macro-activities, namely standing, sitting and walking. It has been evaluated in a static environment and achieves an accuracy rate of 75% in the case of using the K-nearest neighbor (kNN) classifier. The accuracy rate has been improved by using a fusion algorithm and achieves an average accuracy rate of 91%.

#### 2.1.3. RSSI-Based Micro-Activity Recognition

RSSI is used to classify human micro-activities such as hand gestures [40,41] and to estimate human breath rates [42,43]. Melgarejo et al. [40] presented a human hand gesture recognition method which relies on already installed Wireless Local Area Network (WLAN) devices. The proposed method uses the Wireless Open-Access Research Platform (WARP) and was evaluated in two scenarios. It achieves an average accuracy of 92% in a wheelchair scenario with 25 hand gestures and 84% in a gesture-based car information control system scenario.

Abdelnasser et al. [41] presented *WiGest*: the RSSI-based hand gestures recognition method. WiGest uses the fluctuation of RSSI caused by hand motions in the test area. The proposed system classifies a number of gestures with an average accuracy rate of 87.5% when a single access point (a transmitter) is used and 96% when using three overhead access points. In [93], the authors presented a gesture authentication method based on RSSI collected from smart devices (i.e., smartphone and smart watch). However, the proposed method requires a transmitter and receiver to be attached to the target user.

Furthermore, RSSI has been leveraged in breath monitoring. Patwari et al. [42,43] presented an RSSI-based breath monitoring scheme. They showed that chest movements during breathing induces sinusoidal fluctuation in the tested RSSI.

Overall, the RSSI-based device-free mechanism has a significant limitation because of the variability and instability of RSSI induced by changes of testing environments which may cause inaccurate detections.

### 2.2. Wi-Fi Radar

The Wi-Fi radar mechanism has been used in human motion tracking, activity recognition, and hand gesture recognition using Software Defined Radio (SDR). As described above, some of the RSSI and CSI-based sensing approaches used SDR with USRP to track human motion. In this section, we highlight some of the Wi-Fi radar sensing approaches.

Adib et al. presented *WiVi* [94], a radio frequency (RF) based through-wall human motion tracking method by considering human body motion as an antenna array, then tracking the RF beam that resulted from human body motion. *WiVi* has been built by USRP software radios. Adib et al. also presented *WiTrack* [95] and *WiTrack 2.0* [96], device-free methods that track through-wall human motion, which also can recognize simple gestures. They used Frequency Modulated Carrier Wave (FMCW).

In [97], the authors presented a passive Wi-Fi radar to track multiple targets in indoor areas. The key idea of their work is to apply Underdetermined Blind Source Separation (UBSS) to Doppler signal separation of Wi-Fi radar. They proposed a Tree-structured sparse UBSS (TUBSS) method to track several targets in passive Wi-Fi radar. Xie et al. [98] used WARP software defined radio to build a passive or device-free tracking system, namely *xD-Track*. They measured several parameters such as Time of Flight (ToF), Angle of Arrival (AoA), Angle of Departure (AoD), signal attenuation and Doppler shift. The Maximum-Likelihood (ML) was applied to estimate the accurate locations and to detect human motion. The proposed method achieved an average tracking accuracy rate of 98%. Shi et al. [99,100] presented a SDR-based human activity classification system that can recognize activities such as lying, running, standing, crawling, and walking. The proposed activities were implemented in a very restricted area (one to two meter (m) rectangle around the SDR). The SDR is placed next to the room’s door. They proposed a two-stage classifier to detect dynamic actions (i.e., walking) and static actions (i.e., standing), and then classify the proposed activities. The proposed methods achieved an average accuracy of 87%. In [101], a micro-Doppler based HAR method is presented. The proposed method extracts Doppler information from WiFi signals that were reflected by human motion in the test area. Sparse Representative Classifier (SRC) has been applied to classify six activities, namely, pick up from the ground, then stand up, sit on chair, stand up from the chair, fall down, stand up after falling, lie on mattress and then get off mattress. The evaluation performance has achieved an average accuracy rate of 90%. Li et al. [102] presented a micro-Doppler based activity recognition method by applying a multiwindow adaptive S-method which can be applied to analyze the time-frequency of radar signals. They applied the Support Vector Machine (SVM) classifier for six activities and achieved an average accuracy of 95.4%. Additionally, in [103], a micro-Doppler based human motion detection system is presented. The proposed system uses an adaptive S-method and Empirical Mode Decomposition (EMD) to remove non-motion interference. The proposed method can classify several activities and achieved an average accuracy of 97%. Moreover, *WiSee* [104] is a device-free scheme that leveraged the Doppler shift of wireless signals. *WiSee* classifies nine human body gestures that may be applied to contact home wireless connected devices. However, Wi-Fi radar or SDR-based mechanisms have high costs and burdensome installation. In contrast, CSI and RSSI based mechanisms require only ubiquitous WLAN equipment that can be deployed or commissioned easily.

## 3. Channel State Information (CSI)

CSI is considered the new trending metric in Wi-Fi-based sensing technology. Halperin et al. [44,45] presented the CSI-Tool, which can be used to extract CSI from commodity wireless NICs. CSI is the collection of information that describes how wireless signals propagate from the transmitter to the receiver.

To precisely define the CSI, we need some background knowledge about Multiple Input Multiple Output (MIMO) technology.

**Figure 2:**
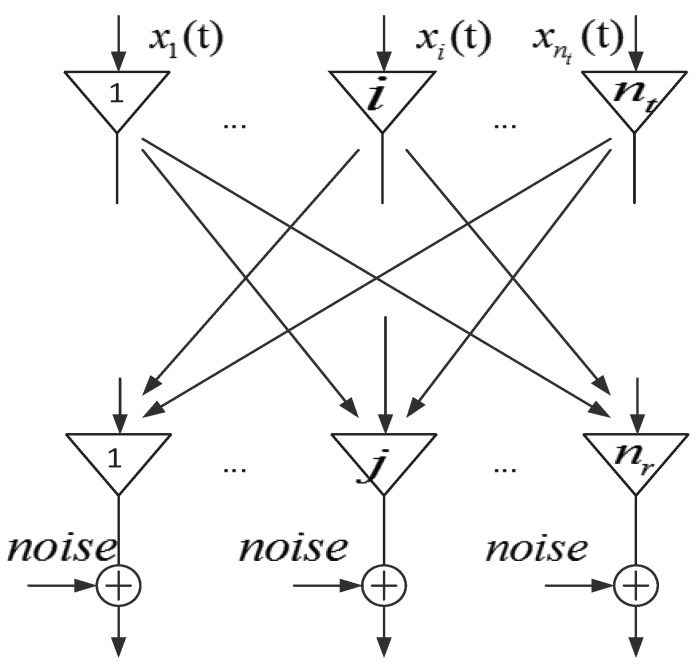
Multiple Input Multiple Output (MIMO) equivalent model.

Figure 2 shows a MIMO equivalent model. The received signal of the *j* antenna can be defined as:

(2) $$y_{j}\left(t\right)=\sum_{i=1}^{n_{t}}h_{i,j}\left(t\right)*x_{i}\left(t\right)+\eta_{j}\left(t\right),i=1,2,\ldots ,n_{t};j=1,2,\ldots ,n_{r},$$

where $H_{(}i,j)$ is the channel fading factor between the transmitted antenna *i* and received antenna *j*. $X_{(}i)$ is the transmitted signals of the antenna *i*. $y_{i}$ is the receive signal of the antenna *j*. Considering the narrowband flat fading channel, Equation (2) can be simplified as:

(3) $$y_{j}\left(t\right)=\sum_{i=1}^{n_{t}}h_{i,j}x_{i}\left(t\right)+\eta_{j}\left(t\right),$$

which can be expressed as:

(4) $$y\left(t\right)=Hx\left(t\right)+\eta \left(t\right),$$

where MIMO system transmit matrix X(t), MIMO system receive matrix y(t), channel additive white Gaussian noise matrix n(t), and channel fading factor matrix *H* are represented as:

(5) $$H=\left[\begin{array}{cccc} h_{1,1} & h_{1,2} & \ldots & h_{1,n_{t}}\\ h_{2,1} & h_{2,2} & \ldots & h_{2,n_{t}}\\ \vdots & \vdots & \ddots \; & \vdots \\ h_{n_{r},1} & h_{n_{r},2} & \ldots & h_{n_{r},n_{t}} \end{array}\right].$$

The CSI uses the channel fading factor matrix *H* that is defined as Equation (5). Each element in the matrix is complex represented [105] as Equation (6):

(6) $$H_{i,j}\left(f_{k}\right)=\left.H_{i,j}(f_{k}\right)e^{\left.j∠H_{i,j}(f_{k}\right)},$$

where $f_{k}$ is the central frequency of the OFDM subcarrier that is defined in the 802.11n protocol, $\left.H_{i,j}(f_{k}\right)$ is the amplitude, and $\left.∠H_{i,j}(f_{k}\right)$ represents the phase shift information.

The most recent device-free human motion and activity recognition studies are based on CSI instead of RSSI because CSI outperforms RSSI in its diversity and stability. Table 2 shows the main differences between CSI and RSSI. CSI is capable of detecting anomalies that occurred due to environmental changes. CSI reflects the varying multipath reflections induced by an intruder’s existence due to its frequency diversity [68].

From the comparison performed in Table 2, it can be seen that CSI is much more stable as compared to RSSI. CSI is adopted to be the future metric of device-free human motion tracking and activity recognition. Some highlights of CSI-based approaches are discussed below.

### 3.1. CSI-Based Localization and Motion Detection

Recently, many studies have been presented by leveraging the CSI for indoor localization and activity recognition. In [49], the authors presented a CSI-based localization system by exploiting CSI variation across OFDM subcarriers. In [50], a CSI-MIMO based localization scheme is presented. The proposed scheme uses the CSI phase and amplitude information of each subcarrier to get the accurate position.

In [106], a human tracking method based on Wi-Fi CSI, namely *WiDar* is presented. The main function of *WiDar* is to estimate the speed and direction of human movement with a relatively velocity error up to 13 cm. Moreover, it can detect a human location with median location errors up to 38 cm without the initial position, and 25 cm with the initial position. The same authors also presented the improved *WiDar 2.0* [107]. *WiDar 2.0* is the subsequent of *WiDar* [106] that harasses multi-dimensional parameters including signals’ attenuation, Time of Arrival (ToA), and Doppler Frequency Shift (DFS). It uses only a single wireless link and achieves an average accuracy of 0.75 m in an area larger than WiDar (6 m × 5 m). Shi et al. [108] presented a CSI-based indoor location tracking system. The proposed system uses principal component analysis (PCA) to remove the noise and a probabilistic model to detect human location. The tracking evaluation achieved average mean absolute errors (MAEs) of 0.63 m when applying a Kalman filter, and 0.17 m in the case of applying a particle filter. In [109], an indoor positioning method based on CSI, namely *MaLDIP*, is proposed. A new subcarrier selection method based on PCA is employed to eliminate multipath affected subcarriers to achieve better localization accuracy. SVM is applied to classify localization and achieved a cell location estimation accuracy of 98.27%. In the case of reduction training samples, it achieved an accuracy of 90%. Soltanaghaei et al. [110] presented a localization system based on CSI called MonoLoco. The proposed system achieved an average localization error of 0.5 m with only one access point and one detection point. Moreover, CSI is exploited to improve BLE localization ability [111].

Xiao et al. [52] presented a CSI-based human motion detection system, namely *Pilot*. The proposed system can effectively detect the presence and location of the target entity. However, it only uses the first antenna of IWL 5300 NIC. Zhou et al. [61,62] modeled the CSI subcarrier amplitude as a histogram, and applied the Earth Mover’s Distances (EMD) algorithm to classify the collected signals. Then, the proposed method builds a fingerprint database and reveals the full range of a human presence in the perceived area.

In [63], the authors leveraged the CSI phase and amplitude information and the spatial diversity provided by MIMO to improve the device-free human motion detection technique. They applied SVM method to determine human motion and designed a passive detection method of a moving human with a dynamic speed, namely *PADS*. The improved version of *PADS* is presented in [112].

Xiao et al. [64] proposed a method of indoor fine-grained motion detection system based on the frequency diversity and time stability of OFDM physical layer CSI. Their proposed system evaluates motion detection by using the normal mode (i.e., CSI mode) in the static environment, proposes a maximum eigenvalue method to extract and characterize the static and dynamic signal mode, and then performs detection of moving behavior by applying a Density-Based Spatial Clustering with Noise (DBSCAN) algorithm. Kianoush et al. [113] presented a CSI-based human motion detection method by investigating the first relevant CSI features that are sensitive to human motion in the test environment. They also proposed a space-frequent selection method by applying PCA. The proposed method can detect two entities motion and localization. Wang et al. [114] presented a device-free CSI-based localization system namely, *LIFS*. LIFS can localize a human target without offline training. The main idea of LIFS is to utilize the subcarriers that are not affected by multipath reflections in the noise environment. The proposed system achieves an accuracy of 0.5 m in the LOS scenario, whereas, in None-line-of-Sight(NLOS), it achieves 1.1 m accuracy. Tian et al. [115] presented a CSI-based indoor localization system. The main idea of this system is to perform frequency domain Crame’r–Rao bound (CRB) for location detection with CSI. The proposed system resolves the high-rank Fisher information matrix challenge and builds an intrinsic connection to estimate the location. The proposed method achieves a localization accuracy of centimeters in different scenarios. Gong et al. [116] proposed an array-based localization system, namely *ROArray*. The proposed system used sparse recovery and coherent processing across time, frequency and spatial domains. The key insights of this system use a combination of the Angle of Arrival (AoA) and the Time of Arrivals (ToA) to detect an object location in an indoor environment. Li et al. [117] proposed an indoor tracking system based on CSI, namely *IndoTrack*. *IndoTrack* is composed of two phases; the first phase is to extract Doppler velocity from CSI, and the second phase is to extract spatial-temporal Doppler and AoA from CSI. The proposed system achieves a trajectory estimation with a 35 cm median error.

Another CSI-based human motion detection system was presented by Gong et al. [118], namely *RFID*. *RFID* can track human motion in indoor environments with a high accuracy rate. The authors proposed two developed real-time detecting methods based on the variation and coefficient of the temporal phase. Additionally, Gong et al. [119] presented CSI-based human detection scheme that can automatically predict the human presence threshold based on the wireless propagation in the test area. The proposed scheme had been evaluated in an indoor environment and achieved a detection rate of 90% in the case of using 30 subcarriers of CSI. In [120], a home intruder detection method had been proposed by using the amplitude of CSI. The authors used wavelet analysis to expose the pattern of motion duration. The proposed method could classify a human through door or window intrusion. In addition, Lv et al. [121] presented CSI-based indoor intrusion detection by leveraging CSI of WiFi. The proposed method namely, *SIED*, detects human motion at different speeds and achieves better accuracy detection in the case of low speed motion. The Hidden Markov Model (HMM) is applied to classify intrusion action.

Yang et al. [122] proposed an occupancy sensing method based on CSI data gathered from IoT Wi-Fi devices. The proposed method can detect a home occupancy and several activities, namely sitting down, jumping, running, swinging a golf club, boxing, and walking. It integrates CSI tools with the OpenWrt system. Then, the collected CSI in IoT Wi-Fi devices should be transmitted to the cloud server. The Singular Value Decomposition (SVD), Nonnegative Matrix Factorization (NMF) and kNN are applied to build the classification model. The proposed method achieved an occupancy detection rate of 96.8%, and 90.6% activities’ classification accuracy rate. Another CSI-based occupancy sensing method is presented in [123].

Wang et al. [124] presented a CSI-based human identification system, namely *DFI*. It exploited CSI intrinsic features by applying empirical mode decomposition (EMD) that decomposes collected CSI into intrinsic mode functions (IMF). *DFI* uses the softmax regression algorithm to identify several human users. The performance of the proposed method achieved 90% of human identification accuracy.

In [125], *Wi-Speed*, a device-free CSI-based object speed estimation method, is presented. *Wi-Speed* estimates a moving object’s speed in an indoor environment and classifies several macro-activities namely, sitting down, picking up, falling down, and standing up. For walk speed estimation, it achieves a mean absolute percentage error of 4.85% and an average detection rate of 95% for fall detection. Furthermore, CSI can be employed to detect car speed in indoor environments as described in [126].

Moreover, Yang et al. [127] presented a sedentary behavior monitoring system using Wi-Fi CSI signals, namely *CareFi*. The main idea of CareFi is to design a foreground detection method to differentiate static and dynamic activities according to their impacts on CSI signals. *CareFi* has been evaluated in two different environments: an office environment and a home environment. Qian et al. [128] presented *WiDance*, a CSI-based system that can track nine whole body dancing motions, which can be applied in human–computer interaction games or other applications. *WiDance* extracts Doppler shifts from the received CSI of Wi-Fi signals to track body motions and directions. The proposed scheme achieved an average accuracy of 92%. The CSI-based technique is also used for crowd counting such as the device-free crowd counting system [67].

### 3.2. CSI-Based Macro-Activity Recognition

In [68], the authors presented *E-eyes*: an indoor CSI-based activity recognition system. They divided human activities into two main categories: the first one is walking activities (i.e., walking). The second category is in-place activities (i.e., phone-call or take a path at the bathroom). Their main idea depends on the fact that each action has a unique histogram distribution of the CSI amplitude. However, the system relies on the position of the implemented activities and does not classify different activities in different positions.

In [69], the authors presented a CSI-based human activity recognition method that analyzes the CSI information at the detection point (the receiver end) of the testing system. The proposed method classifies four macro activities (lying, standing, sitting and walking).

In [70], a human activity recognition method, namely *WIBECAM*, is presented. The proposed method classifies three human activities (standing, sitting, and walking). The main idea of WIBECAM is to collect beacon frames sent by the wireless transmitter periodically. Thus, WIBECAM works as snapshots by monitoring the collected frames and by calculating the frequency domain spectral metrics of each collected frame.

In [71], *CRAM*, a CSI-based activity recognition system is presented. It can classify several human actions, such as pushing, boxing, falling, opening a refrigerator, walking, running, and sitting down. The main idea of *CRAM* is to build a correlation between CSI collected from test environments and the implemented activities. Moreover, several previous literature papers [72,73,74,75] focused on fall detection by using CSI as a sensing metric. Han et al. [72] proposed a CSI-based fall detection method, namely WiFall. The proposed system applied the Local Outlier Factor (LOF) algorithm to detect abnormal data on CSI streams and applied one-class SVM to recognize the fall action. Zhang et al. [74] proposed a CSI-based fall detection method with various fall-like activities in the collected dataset. The phase of CSI is exploited as a salient feature to improve activity classification. Wang et al. [75] presented an improved model of the CSI-based fall detection system by exploring the use of the CSI phase difference between two received antennae. The proposed method was used not only to classify activities, but also to segment a fall action from daily home activities that include both falls and fall-like activities. Ramezani et al. [129] presented a fall detection system by harnessing the CSI of Wi-Fi signals coupled with a ground-mounted accelerometer. Although the human target need not wear or carry devices, the accelerometer is installed in the test area to detect floor vibration. The proposed method reached an accuracy of 95%. Jiang et al. [130] presented an *EI* system, a device-free HAR system based on deep learning. In this study, the authors tried to generalize the device-free technique to implement it in different environments. The proposed EI system classifies six activities, namely wiping the white-board, walking, moving a suitcase, rotating a chair, sitting and standing up and sitting down. In [75], the authors presented a human activity recognition method by applying the LOF algorithm on CSI streams to expose action patterns and applied SVM to classify several human activities. In [76], the authors presented a CSI-based macro activity recognition method based on CSI. They presented a bad-stream elimination method to remove insensitive CSI streams that may lead to false detection. In [77], the authors used PCA to remove the insensitive CSI streams. Palipana et al. [131] presented a CSI-based fall detection method, namely FallDeFi. They considered activities similar to falling such as losing balance, losing consciousness, slipping, and tripping. To detect fall actions, a power burst curve (PBC) has been applied to detect the high frequency events. The SVM has been applied to classify fall actions and achieved a fall detection rate of 93% in the case of a static environment. In the case of environment changes, it achieved an average detection rate of 80%.

Wu et al. [132] presented a through-wall passive CSI-based human activity recognition method, namely TW-See. The proposed method exploits two key techniques to track six human activities, namely walking, sitting down, standing up, falling, hand swing, and boxing. They presented opposite robust PCA (*Or-PCA*) method to obtain the correlation between human activity and CSI value changes, in which the influence of the background environment on the correlation extraction can be eliminated. To detect the start and end times of the human user actions, they presented a normalized variance sliding windows method to segment *Or-PCA* waveforms of the human user actions. They used the back propagation (BP) neural network to classify the proposed activities and achieved a high accuracy rate with 94.46% in the case of the scenario where signals pass through a concrete wall. Li et al. [133] presented Wi-Motion, a human motion detection method based on both amplitude and phase information of CSI. They classified six macro and micro activities namely, bending, hand clapping, walking, making a phone call, sitting down and squatting. The main idea of the performed study is to minimize the phase random offset and using different signal processing methods to obtain a clean dataset. The SVM is applied to classify the proposed six activities and achieves a mean true positive rate of 98.5%.

In [134], *DeepSense*, a CSI and deep learning based HAR method is presented. The autoencoder long-term recurrent convolutional network is used to classify the proposed activities (walk, stand, lie, run, and empty) and achieved a high accuracy rate of 97.4%. Chen et al. [135] presented a CSI-based macro-activity recognition method using deep learning. The proposed method uses bi-directional long short-term memory (ABLSTM) to learn the representative features in two directions from raw CSI. The proposed method classifies six macro-activities, namely falling, walking, sitting down, lying down, standing up, and running. It achieved a high accuracy rate of 97%. In addition, Qing et al. [136] presented a CSI-based HAR method using a random subspace classifier ensemble method.

### 3.3. CSI-Based Micro-Activity Recognition

In [78], the authors presented a hand gesture method based on CSI. The proposed method can recognize four hand gestures in two different scenarios namely, Line-of-sight (LOS) and None-line-of-sight (NLOS). The proposed scheme has achieved an average accuracy of 91% and 89% for LOS and NLOS, respectively. In [79], a hand gesture recognition scheme, namely WiG, is presented. The proposed scheme can classify four hand actions in the LOS scenario with an average accuracy of 92% and in the NLOS scenario with an accuracy of 88%.

In [81], the authors presented a Wi-Fi-based hand gesture recognition method based on CSI, namely *WiGeR*. *WiGeR* applies wavelet analysis and short-time energy (STE) to get the start and end time points of each hand motion. WiGeR achieves an average accuracy rate of 92% in different scenarios with 13 hand gestures. Wang et al. [137] presented a CSI-based gait recognition scheme, namely *WifiU*. *WifiU* had been evaluated with a large dataset of 2800 gesture instances collected from 50 human users walking in an indoor environment. The proposed scheme achieved accuracy rates of 79.28%, 89.52%, and 93.05% for three different scenarios. In [80], an in-air handwriting recognition method has been proposed. The proposed method *WiDraw* uses the AoA of wireless signals at the receiver. The proposed method achieved an average accuracy of 91% for several handwriting motions of several words. In [138], another hand gesture recognition system, namely *WiCatch*, is presented. *WiCatch* can classify nine hand gestures, namely opening the window, waving a hand rightward and leftward, sliding, boxing, pushing, pulling, and opening the fridge. SVM is adopted to classify the proposed method and achieved an accuracy rate of 96%. Fu et al. [139] proposed an in-air hand writing recognition method based on Wi-Fi CSI, namely *Wri-Fi*. The proposed method classifies 26 letters using HMM. *Wri-Fi* achieved average accuracy rates of 86.75% and 88.74% in two different environments. In [140], the authors presented a micro-activity recognition method based on CSI. The proposed methodology uses the Huang–Hilbert transform to detect the start and end times of each activity.

In addition, Zheng et al. [141] leveraged CSI to detect smoking by monitoring different smoking related actions, such as holding, putting up, sucking, putting down, inhaling, and exhaling. The proposed system, namely *Smokey*, was evaluated in an indoor environment with several users and achieved good performance. Moreover, the CSI-based sensing technique can be extended to sense more micro-actions, such as keystrokes [82], finger gestures [142,143], lip motions [83], breath estimation rates and sleeping monitoring. Ali et al. proposed a keystroke recognition scheme based on CSI of Wi-Fi signals, namely *WiKey* [82]. They supposed that as hands type on the keyboard, *WiKey* detects the typed keys because the target user’s hands and fingers create a unique formation and direction and this produces unique patterns in the received CSI.

Li et al. [142] presented a CSI-based finger gestures recognition system, namely *WiFinger*. This system classifies nine-digit gestures (American Sign Language (ASL)) with 90.4% accuracy and achieved 82.67% for individual text inputs for 90 digits. Another finger gesture recognition scheme, also called *WiFinger*, was presented by Tan and Yang [143]. Multi-Dimensional Dynamic Time Warping (MD-DTW) was used to classify different finger gestures. It achieved an average accuracy rate of 93% in two different environments. *WiHear* [83] leveraged fine-grained RF wave information by using a single subcarrier of CSI, partial multipath effect, and discrete wavelet packet transformation to achieve lip reading, and simultaneously classify multiple individuals’ talking using MIMO technology. However, *WiHear* employs specific directional antennae to obtain CSI changes induced by lip motion to recognize several spoken words. *WiSleep* [84] is the first CSI-based method to detect a human respiration rate for sleep monitoring. This study was extended in [85] and several abnormal breathing patterns and sleeping postures were included in the study. The authors in [86] also presented a CSI-based scheme to track the vital signs of human heart rate and breathing rate during sleep. Wu et al. [87] extended the respiration detection from sleeping to standing posture for stationary human detection. Liu et al. [144] presented vital signs and postures during sleep by tracking the fluctuation of CSI caused by minute human body movements. The proposed system can track breathing and heart rates during sleep by applying a breathing cycle and PSD-based K-means clustering methods. It was applied to detect the breathing rate for one and two-persons in bed scenarios. Gu et al. [145] presented Sleepy, a wireless based sleeping monitoring system that can track human micro-motion during sleep. They supposed that the energy features of the CSI follows the Gaussian Mixture Model (GMM) derived from the collected CSI over a long period of time. The proposed system tracks sleeping motions such as rolling over from the background (stationary postures) and achieves an average accuracy of 95.65%.

Furthermore, in [146], a CSI-based method for biometric identification is presented. The proposed method namely BioID tracks simple lip motions to identify different users. BioID uses Dynamic Time Wrapping (DTW) to measure the difference between waveform shapes of CSI, then kNN is used to classify the proposed lip motions. It achieves an average accuracy of 90%. Jia et al. [147] presented WiFind, a device-free system that can be used to detect fatigue with Wi-Fi signals. They built their method based on two modes, breath mode and motion mode. The proposed system applied the Hilbert–Huang transform (HHT) to extract patterns from Wi-Fi signals by tracking driver breath mode to keep track of driver performance. The result of the proposed method achieved 89.6% accuracy with 10% false negative rate (FNR), for a single driver scenario and 73.9% accuracy in a multi-passenger scenario. Another CSI-based driver tracking system, namely ViHOT, is presented in [148].

The readers can follow the recent published studies that used the open-source CSI-Tool in [45].

## 4. CSI Methodology

**Figure 3:**
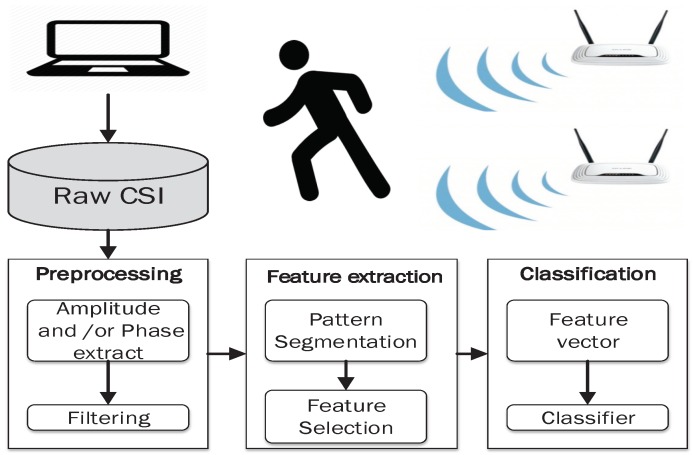
Channel State Information (CSI) based sensing method work flow.

As described in earlier sections, CSI is a potential tool for wireless sensing technology in the future due to its stability and the rich information that can be obtained from the collected packets (per-subcarrier as described in Table 2). Figure 3 shows the general work flow of a Wi-Fi CSI-based sensing mechanism. First, the data collected from CSI-Tool [44], the amplitude, the phase of CSI, or both can be extracted to be analyzed. Second, the collected data are drowned with noise caused by interferences due to the presence of other Wi-Fi channels, and other electromagnetic noise; therefore, filtering is required to get the real trend of CSI. Third, the pattern segmentation and feature extraction methods are applied to build feature vectors as the inputs of the classifier (i.e., a machine learning algorithm, or a deep learning algorithm). Finally, in the classification stage, machine learning or deep learning methods are applied to detect each specific activity or motion.

### 4.1. Preprocessing

In WiFi systems, the chipset modules follow IEEE 802.11n standards to report the CSI to OFDM subcarriers. The 20-MHz bandwidth mode with 64 available subcarriers includes data, pilot, and null subcarriers. However, the IWL5300 Network Interface Card (NIC) is used by the open access CSI-Tool [46], and it only reports CSI data for 30 subcarriers. Therefore, each piece of CSI data contains the number of transmitted antennae $\left(N_{t}\right)$ × number of received antennae $\left(N_{r}\right)$ × 30 subcarriers. In IWL5300 NICs, $N_{r}=3,$ so, by exploiting all three $N_{r}$, the collected data can be calculated as $N_{t}$ × 3 × 30.

**Figure 4:**
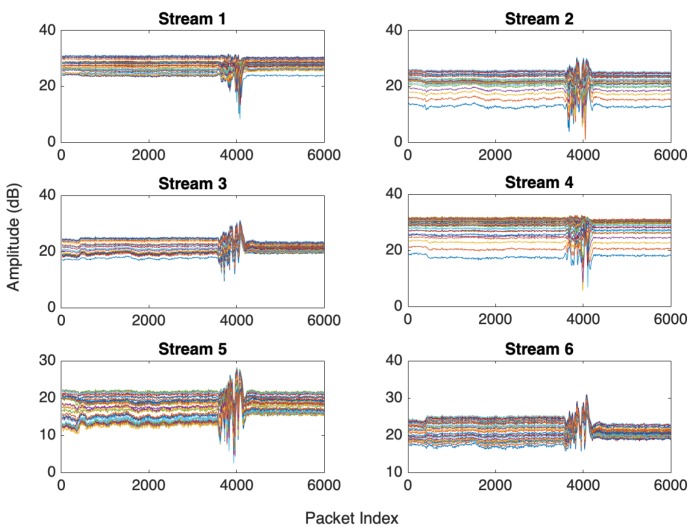
CSI subcarriers of six streams after the filtering of a human fall experiment.

**Figure 5:**
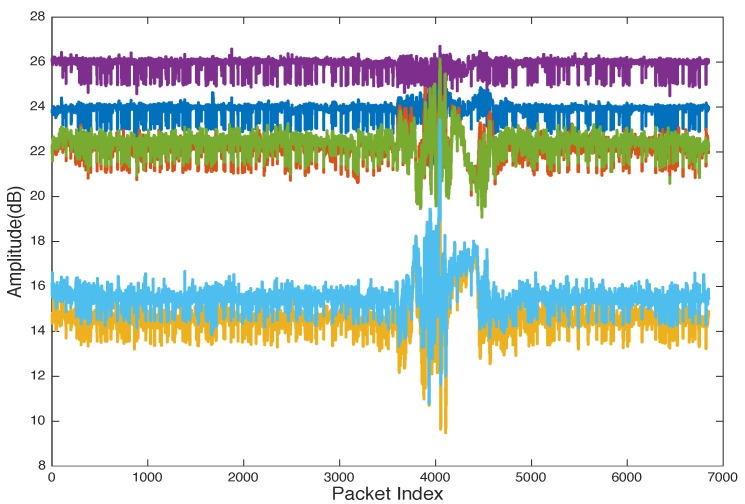
Raw CSI streams of a human through-wall walking experiment.

**Figure 6:**
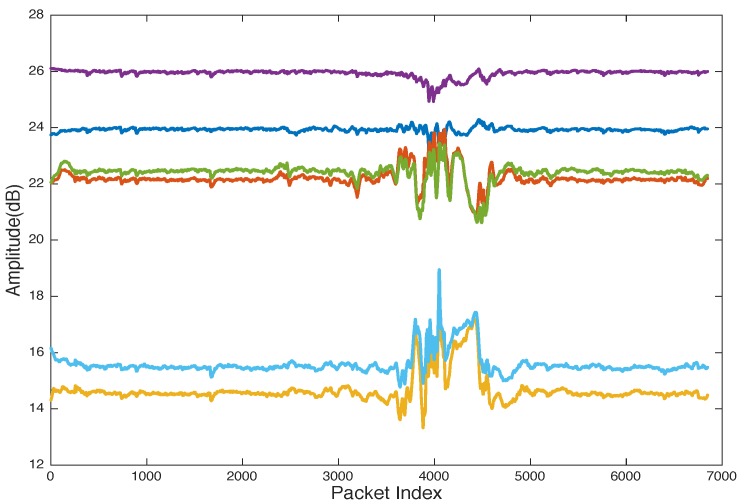
CSI streams of a human through-wall walking experiment after applying exponential filter.

Figure 4 shows the CSI collected from an experiment for a user walking in an indoor environment with a WiFi access point (AP) that has two antennae and a laptop installed CSI-Tool and equipped with IWL5300 NIC with three received antennae as a detection point (DP). The data collected for each transmitted packet can be calculated as 2×3×30. In this experiment, we have six streams, and each stream has 30 subcarriers. To reduce the computation complexity, CSI can be reported as six streams as shown in Figure 5 and Figure 6.

Figure 5 shows that the collected data are drowned with noise from the surrounding wireless devices in the test environments. Many filtering methods can be applied to remove noise such as moving average variance [72], exponential filter [68,77], Butterworth filter [71,81,82], and PCA [71]. Figure 5 shows the six raw CSI streams with noise, and Figure 6 shows the CSI after applying an exponential filter. Figure 6 shows that, after applying the exponential filter, the CSI trend is very clear.

Another problem can be noticed from Figure 6, where CSI streams reported different sensitivities to human motion. Some streams have less sensitivity to human motion, which was termed the “bad antennae” problem [63]. In [120], a bad stream elimination method has been introduced to remove insensitive streams. The bad stream elimination algorithm calculates the max-min, standard deviation, and the mean value of CSI streams and models them as a feature vector to be the input for a Na$\ddot{i}$ve Bayes classifier to detect insensitive streams (bad streams). PCA is also used to remove the bad streams as described in [77].

### 4.2. Feature Extraction

Pattern segmentation and recognition techniques can be used to determine human motion duration. Several techniques have been used for this purpose, such as the Local Outlier Factor (LOF) [72,73,76], wavelet analysis [71], short term energy with wavelet [81,120], envelope extraction [140], and normalized variance sliding windows [132], etc.

**Figure 7:**
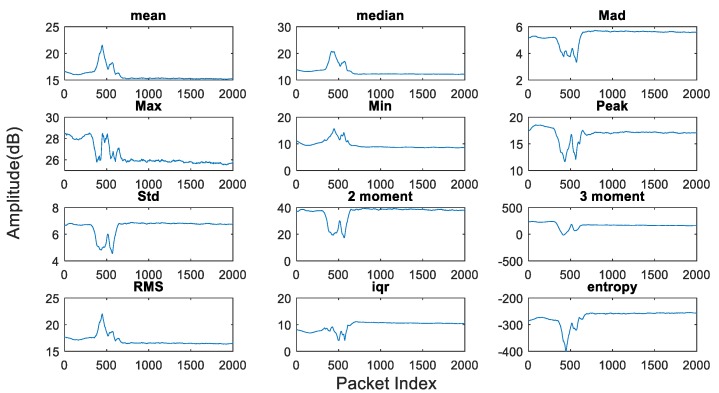
A number of features from CSI time and frequency domains.

Feature extraction can be performed from CSI streams using both the time and frequency domains, such as the mean value, median value, median absolute deviation (MAD), maximum value (Max), minimum value (Min), peak value, normalized standard deviation (STD), second central moment, third central moment, root mean square (RMS), interquartile range (IR) and entropy as shown in Figure 7.

### 4.3. Classification

Various classification methods have been applied to classify human motion in indoor environments such as SVM [63,72,75,120,133], HMM [71], DTW [68,81], kNN and DTW [82], Random Forest (RF) [77], and SRC [69]. Recently, deep learning has also been used to classify human motion based on Wi-Fi signals such as [132]. Table 3 concludes some of the previous CSI-based human motion detection studies. However, almost of the machine learning algorithms and deep learning techniques achieved acceptable detection accuracy and they do not present a challenge in improving device-free Wi-Fi based human motion tracking systems. The most challenging points appear in the pre-processing and pattern segmentation stages.

### 4.4. Test Scenario

**Figure 8:**
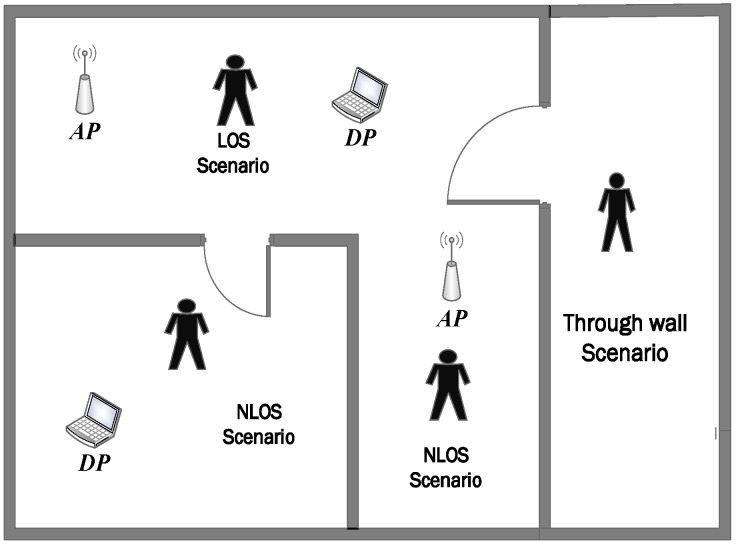
CSI-based sensing scenarios.

The two famous scenarios are the Line-of-Sight (LOS) and None-line-of-Sight (NLOS) as shown in Figure 8. In the LOS scenario, human users implement actions or motion between the AP and the DP. In NLOS scenario, the human user with AP or DP in the LOS scenario, where DP or AP in NLOS. Moreover, another scenario can be considered, namely through-wall scenario where a human user is completely in NLOS with both AP and DP.

## 5. Limitations and Challenges

Wi-Fi CSI-based sensing technology is still immature and requires deeper investigations due to some challenges that need to be addressed in the future. A number of those challenges are listed as follows:

1. **Tracking two or more objects in the test area**. Regardless the recent advancement of CSI-based sensing technology, monitoring two or more objects simultaneously is still a crucial challenge. In [149], the authors presented the first study to address gesture tracking for multiple users. They tested simultaneously performed gestures and studied their impact on wireless signals. Tracking signals for random motions and unfixed orientations is a challenging task and needs to be investigated in literature. Additionally, Ryoo et al. [150] presented *MultiTrack*, a device-free system that can track multiple users activities. However, this system requires each user to perform activities independently to build the signal profile. Therefore, serious efforts are needed to develop technologies in wireless signal processing, MIMO technology, and wireless sensing techniques to cope with these challenges.
2. **Object interference**. Wireless signals are very sensitive to any movement in the test area. Due to the random motion of interfering objects in the test environment, if an object (i.e., humans, pets, etc.) moves in the perceived place, the received signals in the detection part of the Wi-Fi-based sensing system will fluctuate, resulting in difficult detection of human postures, movement, and activity.
3. **Unconstrained mobility**. From previous studies, in the Wi-Fi sensing mechanism, the motion of tested objects is constrained, since wireless signals fluctuated according to object motion in the test area. Therefore, to track an object motion such as a human, the human must move in constrained directions and locations. Hence, building an unconstrained mobility based system may require a deep investigation of wireless signal processing. Additionally, it may require using body sensors and combined sensors with Wi-Fi signals.
4. **CSI universality**. As described earlier, CSI outperforms RSSI in sensing human motion accuracy, but RSSI outperforms CSI in its availability in almost all known WiFi devices. As known, CSI can be extracted only from specific NICs, such as IWL 5300 NICs. There is another CSI-Tool called Atheros CSI-Tool [151] that has been utilized in different CSI-based sensing applications, including localization [152], macro-activity recognition [134], location-independent activity recognition [153], occupancy counting [154], driver activity recognition [155], and gesture recognition [156]. However, Atheros CSI-Tool is also implemented in restricted NICs and operating systems. Thus, RSSI can be applied in many devices, such as smartphones, tablets, or other Wi-Fi devices. This challenge requires great developments in wireless network cards. Recently, Schulz et al. [157] presented a CSI extracted method that can be implemented in smartphones. This method can be used in the future to track human motion based on CSI using smartphones.
5. **Extracted both CSI phase and amplitude information**. Most of the CSI-based sensing methods are used to leverage CSI amplitude information. Just a few previous studies used CSI phase information because the Intel 5300 NICs provided randomly distributed phase information that are always unstable. Therefore, exploiting full phase information might result in improvement of the CSI sensing mechanism to detect more complicated activities.
6. **Environment changes**. Both CSI and RSSI characteristics are not the same for different environments and different people. In indoor environments, wireless signals propagate through multiple paths, such as furniture, floors, and roofs. Therefore, the results of testing a WiFi-based sensing system in an environment may differ in another environment, and, in each new environment, the system classifier needs to be trained again. Moreover, in the presence of a human in the indoor environment, the signal path will experience more fluctuations. Accordingly, different human bodies will cause different variations in the received signals at the receiver. To build a robust Wi-Fi sensing system, environment changes, and different human user bodies and shapes should be considered carefully.
7. **Hybrid sensing methods**. As already discussed in previous sections, different techniques have different limitations; body sensors attached to the user’s body may be used to solve some limitations of current Wi-Fi sensing systems. Therefore, combining body sensors or smartphones with device-free Wi-Fi-based methods into hybrid sensing technologies needs to be addressed in future work. The first simple attempt to combine CSI and wearable devices was presented in [158]. Moreover, CSI can play an important role in the IoT; therefore, hybrid methods to apply CSI in multimedia communications and IoT applications [159,160,161,162] can be addressed. Furthermore, Wireless Sensor Network (WSN) schemes [163,164,165] can be studied.

## 6. Conclusions

In recent years, the recognition of human activities has gained a lot of attention in context-aware research community. Among the areas of its potential applications include Human–Computer Interaction (HCI), surveillance, eldercare, patient monitoring, games, and smart environments. Recently, device-free Wi-Fi-based sensing mechanisms have been presented as the future HAR technology. The device-free mechanisms depend only on Wi-Fi signals; the target object neither requires wearing nor carrying special devices nor is it monitored by particular devices. The Wi-Fi CSI-based sensing mechanism has attracted more attention during the past decade. Although device-free CSI-based sensing techniques have been applied in different sensing applications such as indoor localization, motion detection, and activity recognition, there are still some challenges that need to be addressed. There is a dire need of building reliable CSI-based sensing systems that can effectively track human motion in more complex scenarios. Therefore, by applying more solutions to the current challenges, we believe that the Wi-Fi CSI-based sensing techniques can be considered as the future backbone of HCI.

## Figures and Tables

**Table 1:** Review on device-based human activity recognition systems.

Literature	Device	Drawbacks
[5,6]	Camera	Requires good light conditions, and cannot go through a wall.
[7,8]	Acoustic sensors	Require carrying or installing acoustic sensors.
[9,10]	Accelerometer sensors	Require a human to carry devices supplied with accelerometers.
[11,12]	Wearable sensors	Require a human to wear body sensors.
[13,14]	Environment installed sensors	Require heavy installation.
[15,16]	Smartphone	Requires a human to carry a smartphone.

**Table 2:** A comparison between Channel State Information (CSI) and Received Signal Strength Indicator (RSSI).

Metric	RSSI	CSI
Network layer	MAC layer	Physical layer
Time resolution	Packet size	Multipath signal cluster scale
Frequency resolution	No	Subcarrier scale
Temporal stability	Low	High
Measurement band	RF band	Base band
Granularity	Coarse-grained (per packet)	Fine-grained (per subcarrier)
Universality	Almost all Wi-Fi devices	Some Wi-Fi devices

**Table 3:** Some of the classifiers that are used to classify CSI-based human motion detection.

Literature	Type of Classified Motion	Classifier	Performance
FIFS [48]	Human localization	Probability model	Achieves a mean error lower than 1 m
CSI-MIMO [50]	Human localization	Deterministic kNN and the probabilistic Bayes rule	Achieves an accuracy of 0.95 m
PADS [63]	Human motion	SVM	Achieves a true positive rate of 94%
E-eyes [68]	In-place activity: empty, cooking, eating, washing dishes, studying, brushing, bathing.	Earth mover’s distance (EMD) and Multi-Dimensional Dynamic Time Warping (MD-DTW)	Achieves 96% average true positive rate;
CRAM [71]	Walking, running, sitting down, falling, opening refrigerator, boxing, brushing teeth, pushing one hand, and empty	Model activities with Hidden Markov Model (HMM), and using highest likelihood to identify the activity	Achieves an accuracy of 96%
WIBECAM [70]	Empty, walking, standing, sitting	Linear discriminant analysis	Achieves accuracies from 0.73 to 1 in different environments
Wei et al. [69]	Walking, standing, lying, and sitting	Sparse Representative Classifier (SRC)	Achieves 90% of accuracy in case of Window size = 10;
EI [130]	Wiping the white-board, walking, Moving a suitcase, rotating the chair, sitting, standing up and sitting down	Convolutional Neural Network (CNN)	Achieves an accuracy of 0.75 with balance constraint and 0.6 without balance constraint
Wu et al. [132]	Walking, sitting down, standing up, falling, hand swing, and boxing	BP neural network	Achieves an accuracy rate of 94.46%
Li et al. [133]	Bend, and hand clap, walk, phone call, sit down and squat	SVM	Achieves a mean true positive of 98.5%.
WiFall [72]	Fall detection	SVM	90%
RT-Fall [75]	Fall detection	SVM	Achieves an accuracy of 100%
Li et al. [77]	Sit down, lie, walk, squat down, stand up, crawl, and fall	RF	Achieves a detection accuracy of 95% in LOS, and 91% in NLOS
WiG [79]	4 hand motions (left, right, up, down)	SVM	Achives an accuracy of 93%
WiCatch [138]	Open the window, boxing, open the fridge, push, pull, slide, leftward, rightward, and wave hand;	SVM	Achieves an accuracy of 96%
WIHEAR [83]	Lip motion for Several world syllabus	DTW	Achieves an accuracy of 91% for one individual speaking 6 words; and 74% for 3 people speaking simultaneously.
WiKey [82]	37 Keystrokes (10 digits, one space bar and 26 letters of the alphabets))	kNN classifier	Achieves keystrokes detection rateof 97.5% and 96.4% of a single key accuracy rate.
WiFind [147]	Detect driver fatigue by tracking human body breath and motion.	SVM	Achieves an accuracy of 89.6% for single driver;
Sleepy [145]	Sleep monitoring (tracing human motion during sleep)	Probability model	Achieves 95.65% detection accuracy
